# Single-Genotype Syntrophy by *Rhodopseudomonas palustris* Is Not a Strategy to Aid Redox Balance during Anaerobic Degradation of Lignin Monomers

**DOI:** 10.3389/fmicb.2016.01082

**Published:** 2016-07-14

**Authors:** Devin F. R. Doud, Largus T. Angenent

**Affiliations:** Department of Biological and Environmental Engineering, Cornell UniversityIthaca, NY, USA

**Keywords:** single-genotype syntrophy, *Rhodopseudomonas palustris*, microbial electrochemistry, lignin degradation, redox balance

## Abstract

*Rhodopseudomonas palustris* has emerged as a model microbe for the anaerobic metabolism of *p*-coumarate, which is an aromatic compound and a primary component of lignin. However, under anaerobic conditions, *R. palustris* must actively eliminate excess reducing equivalents through a number of known strategies (e.g., CO_2_ fixation, H_2_ evolution) to avoid lethal redox imbalance. Others had hypothesized that to ease the burden of this redox imbalance, a clonal population of *R. palustris* could functionally differentiate into a pseudo-consortium. Within this pseudo-consortium, one sub-population would perform the aromatic moiety degradation into acetate, while the other sub-population would oxidize acetate, resulting in a single-genotype syntrophy through acetate sharing. Here, the objective was to test this hypothesis by utilizing microbial electrochemistry as a research tool with the extracellular-electron-transferring bacterium *Geobacter sulfurreducens* as a reporter strain replacing the hypothesized acetate-oxidizing sub-population. We used a 2 × 4 experimental design with pure cultures of *R. palustris* in serum bottles and co-cultures of *R. palustris* and *G. sulfurreducens* in bioelectrochemical systems. This experimental design included growth medium with and without bicarbonate to induce non-lethal and lethal redox imbalance conditions, respectively, in *R. palustris*. Finally, the design also included a mutant strain (NifA^*^) of *R. palustris*, which constitutively produces H_2_, to serve both as a positive control for metabolite secretion (H_2_) to *G. sulfurreducens*, and as a non-lethal redox control for without bicarbonate conditions. Our results demonstrate that acetate sharing between different sub-populations of *R. palustris* does not occur while degrading *p*-coumarate under either non-lethal or lethal redox imbalance conditions. This work highlights the strength of microbial electrochemistry as a tool for studying microbial syntrophy.

## Importance

Synthrophic microbial relationships are of utmost importance in nature. They resolve important electron flow issues under anaerobic conditions and make microbial life under difficult thermodynamic conditions possible. During the anaerobic breakdown of the electron-rich aromatic ring in monomers of lignin, the photoheterotrophic bacterium *R. palustris* must strategically dispose of excess reducing equivalents. Researchers had in the past hypothesized that a syntrophy may exist within a single culture—cells that convert the monomer into acetate and other cells that further oxidize acetate. However, conclusive proof is elusive. Here, we used a co-culture of *R. palustris* and *Geobacter sulferreducens* in a bioelectrochemical system to examine if such a single-genotype syntrophy exists. A 2 × 4 experimental design with several positive controls under both non-lethal and lethal conditions did not identify any evidence for a single-genotype syntrophy strategy by *R. palustris*, failing to provide support for this hypothesis.

## Introduction

Lignin is the most abundant source of organic aromatic compounds, and the second most abundant organic carbon source in the biosphere (Suhas et al., 2007). Due to its overwhelming supply, lignin presents itself as a prime substrate for biomass conversion to produce renewable resources and energy. Unfortunately, the abundance of lignin is rivaled only by the difficulty microbes have in metabolizing it, especially under anaerobic conditions (Beckham et al., 2016). This is because lignin is a class of structurally complex, high molecular weight molecules that contain many aromatic groups and remain largely insoluble. Previous studies on anaerobic lignin metabolism have focused primarily on the degradation of lignin monomers and the demonstration of anaerobic aromatic ring fission (Evans, 1963; Healy and Young, 1979; Colberg and Young, 1982; Porter and Young, 2013). Since this discovery, degradation of lignin oligomers and monomers have been demonstrated under strictly anaerobic conditions, though, it is still debated if complex lignin can be degraded under these conditions (Kirk and Farrell, 1987; Brown and Chang, 2014).

*Rhodopseudomonas palustris* is a model microbe for lignin monomer degradation and has also emerged as an attractive microbe for bioenergy production. This purple non-sulfur bacterium has been well-characterized for its anaerobic metabolism of lignin monomers (e.g., *p*-coumarate) in the presence of light (Harwood and Gibson, 1986; Pan et al., 2008; Hirakawa et al., 2012; Phattarasukol et al., 2012). However, while growing photoheterotrophically with *p*-coumarate, *R. palustris* must orchestrate a number of metabolic strategies for managing excess reducing equivalents that accumulate from this aromatic substrate. While CO_2_ fixation and H_2_ evolution have both been previously implicated in managing the redox balance for *R. palustris* (McKinlay and Harwood, 2010, 2011), determining whether every cell is performing these redox-balancing activities, or if it is a shared strategy between an entire community, has remained obscure.

It has been hypothesized that while degrading *p*-coumarate in pure culture, *R. palustris* forms a pseudo-consortium with division of metabolic tasks (1. *p*-coumarate to benzoate, 2. benzoate to acetate/formate/H_2_, and ultimately 3. acetate/formate/H_2_ oxidation) between sub-populations, resulting in a single-genotype syntrophy (Karpinets et al., 2009). The observation that *R. palustris* releases acetate when growing with *n*-butyrate provides support that acetate could be a preferred metabolite to share when growing on a variety of reduced substrates (McKinlay and Harwood, 2011). By secreting acetate during *p*-coumarate degradation to be utilized by another sub-population, this strategy reduces the redox imbalance that would arise within a single cell converting *p*-coumarate completely to CO_2_. We refer to this here as acetate sharing. This reduces the demand for electron acceptors within the *p*-coumarate-degrading population, and thus enables the redox balance to be shared between the members in this hypothesized pseudo-consortium. Due to the metabolic versatility of the *R. palustris* genome, acetate sharing would employ many of the same thermodynamic advantages that are present within a complex microbial consortium.

Complications in experimentally verifying acetate sharing exist since transcriptomic or proteomic analyses from bulk pure culture studies measure all sub-populations together as an average, and many limitations still exist with bacterial single-cell RNA-seq (Saliba et al., 2014). To experimentally test whether *R. palustris* utilizes acetate sharing as a redox strategy while metabolizing *p*-coumarate, we coupled *R. palustris* with the acetate-oxidizing, electrochemically active microbe *Geobacter sulfurreducens* within a bioelectrochemical system (BES). In this system, *G. sulfurreducens* functions as a surrogate for the hypothesized sub-population of *R. palustris* that is responsible for acetate oxidation, and as a reporter for the magnitude of acetate sharing.

*G. sulfurreducens* is a model microbe for a high efficiency conversion of both acetate and H_2_ into electric current when grown at an oxidizing electrode (anode) in a BES (Bond and Lovley, 2003). Conserving energy for metabolism in the process, *G. sulfurreducens* completely oxidizes both acetate and H_2_ with electrons exiting the system through an electrical circuit of a BES, resulting in an electric current. By complementing *R. palustris* growing on *p*-coumarate with *G. sulfurreducens* in a BES under conditions that present a challenge to cellular redox, an anaerobic co-culture of these two microbes could probe whether acetate sharing within a single-genotype population of *R. palustris* occurs. Therefore, the electric current (real-time output signal) from the reporter strain *G. sulfurreducens* serves as a proxy for acetate sharing by the *p*-coumarate degrading subpopulation of *R. palustris*. Because of the ability of *G. sulfurreducens* to channel electrons out of the system *via* the electrode, and since a buffer would neutralize excess H^+^, *G. sulfurreducens* functions as a sink for excess reducing equivalents in the form of acetate or H_2_. Further, acetate oxidized by *G. sulfurreducens* can return back to *R. palustris* in the form of CO_2_, even when exogenous ${\text{HCO}}_{3}^{-}$ is omitted from the media. Therefore, if *R. palustris* engages in acetate sharing, a BES co-culture between *R. palustris* and *G. sulfurreducens* would ease the burden of redox imbalance, and could even rescue growth of *R. palustris* from conditions that would otherwise induce a lethal redox imbalance (−${\text{HCO}}_{3}^{-}$).

Our 2 × 4 experimental design for *p*-coumarate degradation included with and without bicarbonate in the growth medium (Table 1). With bicarbonate, *R. palustris* CGA009 has the exogenous electron acceptor CO_2_ available to get rid of reducing equivalents by fixing CO_2_ into biomass and allowing growth by avoiding lethal redox imbalance (1 in Table 1). Without bicarbonate, and in the presence of ${\text{NH}}_{4}^{+}$, wild-type *R. palustris* CGA009 does not have enough electron acceptors to maintain redox balance, resulting in arrested growth (McKinlay and Harwood, 2010) (2 in Table 1). The study was designed based on four strain combinations and the experimental design included the mutant strain (NifA^*^) of *R. palustris* CGA009, which constitutively expresses nitrogenase genes in the presence of ${\text{NH}}_{4}^{+}$ to secrete H_2_. H_2_ production reduces the strain of reducing equivalents in a way to ensure a non-lethal redox imbalance condition even without bicarbonate in the growth medium. It, therefore, serves as a redox-balanced positive control for without bicarbonate experiments (3 and 4 in Table 1).

**Table 1:** **2 × 4 experimental design for *p*-coumarate degradation with *R. palustris* and two additional positive controls with different substrates**.

**Substrate**	**Culture**	***R. palustris* strain**	**+${\text{HCO}}_{3}^{-}$**	**−${\text{HCO}}_{3}^{-}$**
*p*-Coumarate	Pure culture	Wild-type *R. palustris* CGA009	1	2
		NifA^*^-mutant *R. palustris*	3	4
	Co-culture with *G. sulfurreducens*	Wild-type *R. palustris* CGA009	5	6
		NifA^*^-mutant *R. palustris*	7	8
*n*-Butyrate		Wild-type *R. palustris* CGA009	9	−
Acetate		NifA^*^-mutant *R. palustris*	−	10

**Figure 1:**
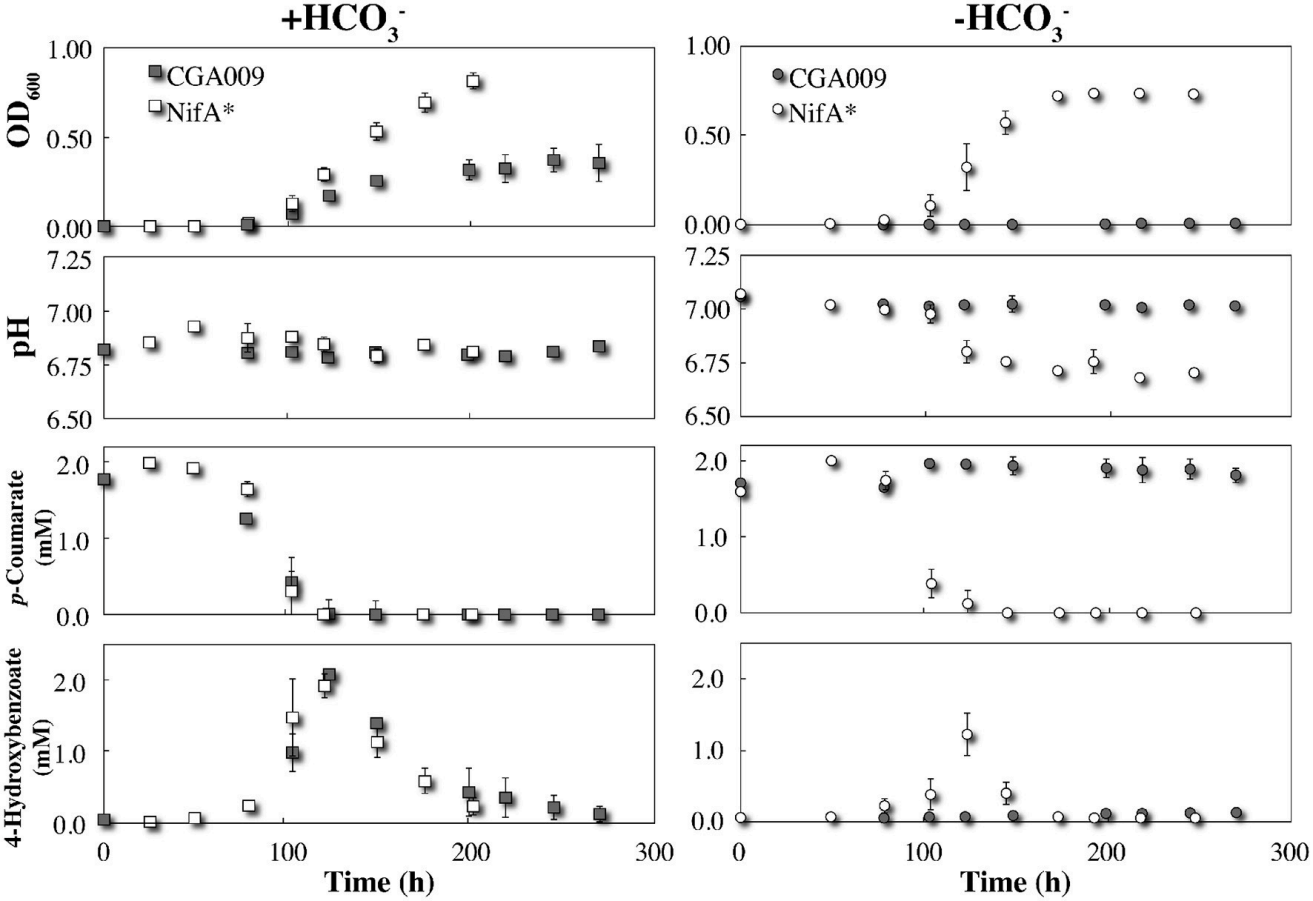
Triplicate growth of *R. palustris* strains CGA009 and NifA^*^ in FW medium 2 mM *p*-coumarate with and without bicarbonate (${\text{HCO}}_{3}^{-}$). Squares and circles represent +${\text{HCO}}_{3}^{-}$ and −${\text{HCO}}_{3}^{-}$ conditions, respectively. Gray icons denote *R. palustris* CGA009 and white icons denote *R. palustris* NifA^*^. All precultures for growth experiments were in FW 2 mM *p*-coumarate with bicarbonate medium except for the NifA^*^ culture, which was grown without bicarbonate to avoid the log lag phase when adapting from with to without bicarbonate conditions.

*Numbers in the final two columns are identified in the text. Results are shown for: 1–4 in Figure 1; 5 and 7 in Figure 3; 6, 8, and 10 in Figure 4; and 9 in Figure 5*.

Non-lethal 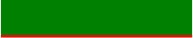

Lethal 
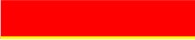

Non-lethal with electric current reporter 
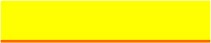

Lethal with electric current reporter 
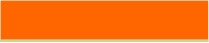

Non-lethal, electric current positive. 
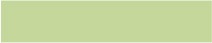

For the co-culture experiments in BESs with the reporter strain *G. sulfurreducens*, the wild-type *R. palustris* CGA009 cannot produce H_2_. Thus, any electric current generation with this co-culture is indicative of acetate sharing under a non-lethal redox imbalance condition with bicarbonate (5 in Table 1). Importantly, electric current generation for the more stringent, lethal redox imbalance condition without bicarbonate would strongly be indicative of acetate sharing because CGA009 cannot otherwise grow under these conditions (6 in Table 1). Finally, for the co-culture experiments with the NifA^*^ mutant, we anticipate electric current production even without acetate sharing because *G. sulfurreducens* can consume the H_2_ secreted by NifA^*^, thus functioning as a positive control for *G. sulfurreducens*-produced current through consuming metabolites (H_2_) shared by *R. palustris* (7 in Table 1). However, without bicarbonate we do not anticipate an electric current without acetate sharing. While *G. sulfurreducens* can generate current from acetate in the absence of CO_2_(Sun et al., 2014), the behavior of *G. sulfurreducens* is poorly understood in the absence of CO_2_ (Soussan et al., 2013), and activity with H_2_ has never been reported. Thus, any sustained electric current would be indicative of acetate sharing with NifA^*^ in this co-culture (8 in Table 1).

In addition to these eight (2 × 4) experiments for *p*-coumarate degradation with *R. palustris* under anaerobic conditions, we performed two additional experiments with other substrates to function as positive controls for acetate sharing with the reporter strain *G. sulfurreducens*. The substrate *n*-butyrate was a positive control to show that, in principle, acetate sharing would be possible under non-lethal redox imbalance conditions for the wild-type strain CGA009 (9 in Table 1). In addition, acetate was fed directly to the NifA^*^ + *G. sulfurreducens* co-culture without bicarbonate to validate the reporter strain (*G. sulfurreducens*) was active and to rule out that a negative result (no current) was indeed an indication for the absence of acetate sharing rather than a problem with the reporter strain or equipment (10 in Table 1). In summary, this study was designed to conclusively ascertain whether acetate sharing occurs within a single-genotype consortium of *R. palustris* while degrading *p*-coumarate for maintaining redox balance at two stringency levels: non-lethal and lethal redox imbalance conditions.

## Materials and methods

### Growth

*Rhodopseuomonas palustris* strains CGA009 and NifA^*^, which were provided by Dr. Caroline Harwood (University of Washington), and *Geobacter sulfurreducens* were routinely cultivated in filter-sterilized anaerobic fresh water (FW) medium. FW medium consisted of 2.5 g NaHCO_3_, 0.1 g KCl, 0.25 g NH_4_Cl, 0.52 NaH_2_PO_4_, 10 mL FW Vitamins, and 1 mL FW Minerals per liter, which was neutralized to pH 7.0 (Li et al., 2012). Precultures of *G. sulfurreducens* were grown in FW medium with 10 mM acetate and 20 mM fumarate acting as carbon substrate and terminal electron acceptor, respectively. Precultures of *R. palustris* CGA009 and NifA^*^ were grown in the light with 2 mM *p*-coumarate as the sole organic carbon source. NaHCO_3_ was replaced with a 25 mM phosphate buffer at a final pH of 7.0 for precultures of NifA^*^ intended for experiments in the absence of ${\text{HCO}}_{3}^{-}$. Toxicity screens validated that 2 mM *p*-coumarate did not inhibit any of the strains used in this study (data not shown).

### *R. palustris* characterization in serum bottles

Batch serum bottle experiments for the characterization of CGA009 and NifA^*^ strains in pure culture with *p*-coumarate in FW media with and without bicarbonate were incubated in triplicate in an environmental growth chamber (GC8-2VH, EGC, Chagrin Falls, OH). Conditions were maintained at 30.0°C with 80 μmol of photons/s/m^2^ (photons between 400-700 nm) from both fluorescent and incandescent lamps.

### Bioelectrochemical reactors

Two-chamber, H-type reactors were used for all electrochemical experiments. The reactors were constructed out of autoclavable glass with water jackets for temperature control and ports for electrochemical components (TerAvest et al., 2014). The working electrode consisted of 9 × 9 cm carbon cloth (PANEX ® 30 − PW06, Zoltek Corp, St Louis, MO), which was attached to a carbon rod with carbon cement (CCC Carbon Adhesive, EMS, Hatfield, PA). The working electrode was potentiostatically controlled (VSP, BioLogic USA, Knoxville, TN) at +0.300 V vs. Ag/AgCl using an Ag/AgCl/sat'd KCl reference electrode (made in-house). The counter electrode consisted of a 2 × 7 × 1 cm carbon block (Poco Graphite, Decatur, TX), which was attached to a carbon rod with carbon cement (CCC Carbon Adhesive) and was separated from the counter chamber by a cation exchange membrane (Membranes International, Ringwood, NJ). Prior to the operating period, the reactors were autoclaved and only sterile components were added. The working chamber contained 450 mL of FW medium and the counter chamber contained 450 mL of FW medium with no carbon source. The reactors were maintained at 30.0°C with water jackets and a recirculating water heater (Model 1104, VWR Scientific, Radnor, PA) and uniformly illuminated with 60-W incandescent lamps at an intensity of ~ 40 W/m^2^. Lids with butyl rubber stoppers were used to maintain gastight conditions while sampling and replacing the medium. Anaerobic conditions were maintained by sparging reactors with either 80:20 N_2_/CO_2_ or N_2_ through sterile filters for conditions with and without bicarbonate, respectively.

### Electrochemical experiments

For co-culture experiments, *G. sulfurreducens* was initially grown at the anode in FW medium with 10 mM acetate under continuous-flow conditions in biological triplicates. After a biofilm and stable current production were achieved, the working chamber was flushed at a rate of 0.75 L/h with 1.5 L of either sterile anaerobic FW medium with 2 mM *p*-coumarate and: (1) 30 mM ${\text{HCO}}_{3}^{-}$; or (2) a 25 mM phosphate buffer and no ${\text{HCO}}_{3}^{-}$. The reactors were then operated in batch with no sparging for the conditions with ${\text{HCO}}_{3}^{-}$ and active N_2_ sparging for the conditions without ${\text{HCO}}_{3}^{-}$. Following media replacement, the reactors were allowed to reach an electrical baseline before inoculating them with *R. palustris*. Samples were taken throughout the operating period to monitor OD_600_, pH, and relevant metabolites. All non-aromatic metabolites were detected *via* HPLC (600 HPLC, Waters, Milford, MA) with a refractive index detector and an Aminex HPX-87H column (Bio-Rad, Hercules, CA). The column was maintained at a temperature of 60°C, and a 5 mM sulfuric acid eluent at a flow rate of 0.6 mL/min was used as the mobile phase. Aromatic metabolites were detected *via* a Thermo Scientific Ion Chromatograph System (ICS-1100, Dionex, Sunnyvale, CA) with a Dionex IonPac™ AS22 column (4 × 250 mm) and a Dionex Variable Wavelength Detector set to 285 nm. AS22 eluent was used at a flow rate of 1.2 ml/min. Metabolites were identified by retention times from high purity standards (Sigma-Aldrich, St. Louis, MO).

### Microscopy

Mid-log phase cultures of *R. palustris* CGA009 and NifA^*^ growing in batch serum bottles containing FW 2 mM *p*-coumarate with ${\text{HCO}}_{3}^{-}$ were visualized using a KH-7700 digital microscope system (Hirox, Hackensack, NJ). Liquid samples were removed from growing cultures, 10 μL was added directly to a microscope slide with coverslip, and was directly visualized from above with a Hirox MX(G)-10C OL-140II lens. Cell aggregate geometries were measured using the integrated Hirox software and approximately 50 measurements were averaged.

## Results and discussion

### *R. palustris* strains CGA009 and NifA^*^ show similar metabolic profiles while degrading *p*-coumarate with bicarbonate under anaerobic conditions

We inoculated the wild-type *R. palustris* CGA009 into serum bottles containing anaerobic FW medium with 2 mM *p*-coumarate with and without the addition of ${\text{HCO}}_{3}^{-}$. As mentioned above and described in the literature (McKinlay and Harwood, 2010), the wild-type *R. palustris* CGA009 was only able to degrade *p*-coumarate and grow when bicarbonate was added to the anaerobic medium to avoid a lethal redox imbalance (Figure 1). The mutant *R. palustris* NifA^*^, on the other hand, was able to degrade *p*-coumarate and grow with and without bicarbonate due to the formation of H_2_ and the resulting elimination of excess reducing equivalents (Figure 1).

The pathway for *p*-coumarate metabolism by *R. palustris* has been previously studied with identification of all intermediates and mechanisms (Pan et al., 2008). To ascertain whether any major metabolic differences between strains CGA009 and NifA^*^ exist, we carefully compared their behavior under identical conditions in serum bottles with 2 mM *p*-coumarate under anaerobic conditions. We observed that the metabolism of *p*-coumarate by *R. palustris* occurs in distinctive phases, starting with the non-β-oxidative cleavage of the alkyl side chain, and yielding a nearly stoichiometric conversion to 4-hydroxybenzoate (Figure 1). The observation that *p*-coumarate is converted entirely to 4-hydroxybenzoate at a 1:1 ratio suggests no sub-population is degrading downstream metabolites and the only active metabolism at that time point is on the alkyl side chain. The hydroxyl group was then removed to produce benzoate (not detected), which was rapidly degraded by β-oxidation after activation and cleaving the aromatic ring (Harrison and Harwood, 2005; Pan et al., 2008). The transient production of 4-hydroxybenzoate began with the onset of *p*-coumarate metabolism and disappeared with the plateau of maximum culture OD, suggesting it is rapidly consumed once taken up by the cell (Figure 1). No other downstream metabolites, including acetate, were detected *via* HPLC. With bicarbonate, *R. palustris* CGA009 and NifA^*^ both have very similar rates of metabolism of *p*-coumarate and 4-hydroxybenozate, resulting in similar consumption profiles. This similar metabolic behavior for CGA009 and NifA^*^ under anaerobic conditions is helpful for the rest of our comparative study.

Despite nearly identical rates of substrate consumption, the optical density between the two cultures rapidly diverged with *R. palustris* NifA^*^ reaching a density more than double that of CGA009 (0.372 ± 0.067 vs. 0.847 ± 0.002 maximum OD_600_; Figure 1). This result is counterintuitive, however, because CGA009 should produce more biomass per unit of substrate consumed compared to NifA^*^ due to the extra reducing equivalents available to CGA009 to fix CO_2_ that are not lost to H_2_ evolution (McKinlay and Harwood, 2010). The observation that substrate consumption still occurred at the same rate within both environments, however, suggests that a similar level of active cells must be present within both cultures.

**Figure 2:**
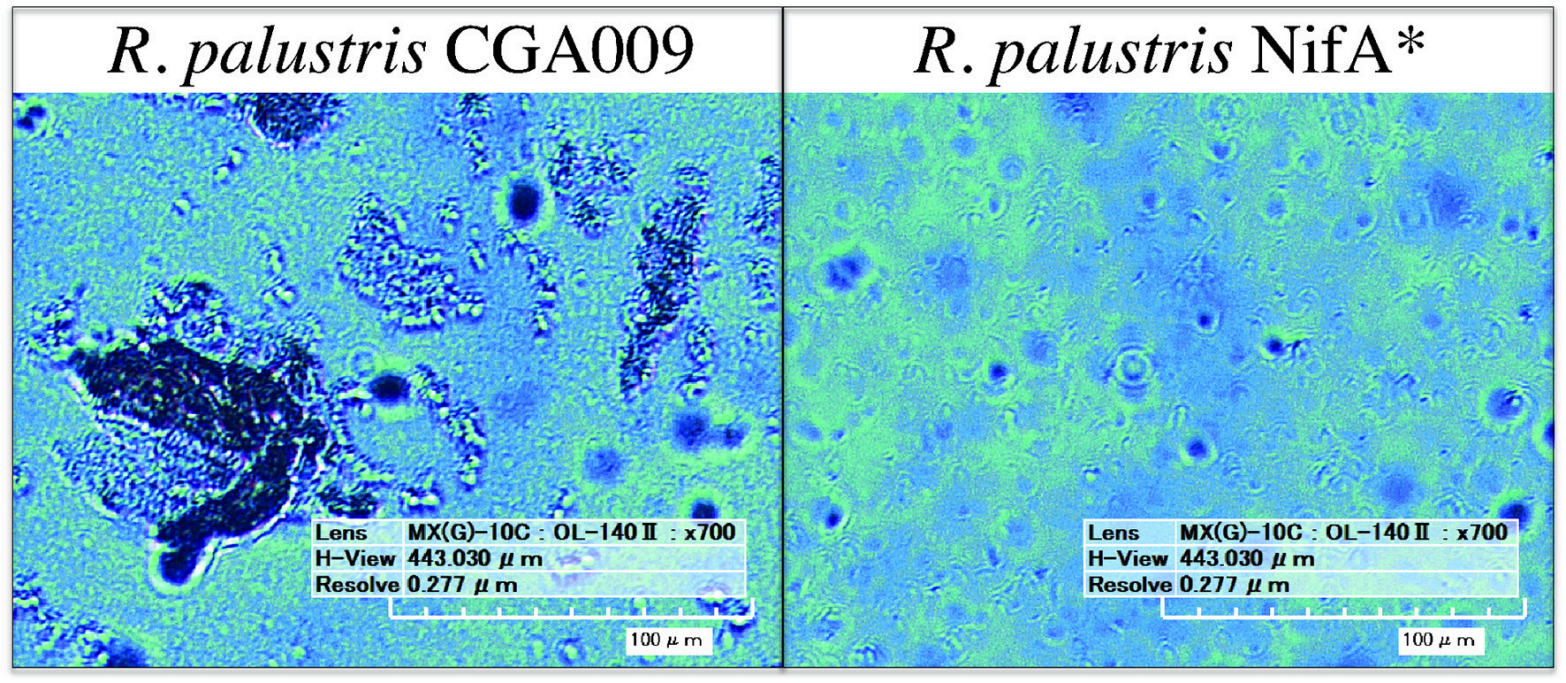
Digital microscopy of *R. palustris* CGA009 and NifA^*^ grown in pure culture on FW 2 mM *p*-coumarate with bicarbonate. Aggregates of cells appear as dark clumps (CGA009), single cells can be seen as ~ 4 μM long rods (NifA^*^).

To investigate this, digital microscopy of the two cultures revealed that under these conditions CGA009 tended to grow primarily in granule-like aggregations ranging from 20 to 200 μm in width, while NifA^*^ grew primarily as single cells with only infrequent small granules observed (Figure 2). The reduced optical density of CGA009 compared to NifA^*^ is, thus, likely due to aggregation under this condition as substrate metabolism rates demonstrate that similar levels of active cells were likely present, while optical densities varied between the two cultures. Because the only difference between strain CGA009 and NifA^*^ is the constitutive expression of nitrogenase genes in NifA^*^, this phenotypic distinction can be attributed to the alternate pathways for maintaining redox balance (i.e., CO_2_ fixation vs. H_2_ evolution) and demonstrates the drastically different outcomes that these strategies can have on global cellular behavior. This observation further suggests that metabolite sharing could be a viable strategy undertaken by a CO_2_-fixing pseudo-consortium of *R. palustris* CGA009 due to the tendency to aggregate, since this aggregating phenotype is abolished when H_2_ secretion by individual cells is utilized as the primary route to redox balance.

### Acetate sharing under non-lethal redox imbalance conditions was not observed

To investigate whether acetate sharing occurs under non-lethal redox conditions, *R. palustris* CGA009 and NifA^*^ were independently inoculated into *G. sulfurreducens*-pregrown BESs containing 2 mM *p*-coumarate as the sole carbon source in FW medium with bicarbonate. Because both strains of *R. palustris* are capable of growing under these conditions, the presence of *G. sulfurreducens* on the electrode as a potential electron sink is not obligate for growth for either strain under these conditions and any current produced would be through metabolite sharing of either acetate (CGA009 or NifA^*^) or H_2_ (limited to NifA^*^). Since acetate sharing is proposed as a strategy that develops in pure cultures, the *R. palustris* precultures used to inoculate the electrochemical reactors would already be performing acetate sharing, minimizing any adaptation response upon introduction into the BES and eliminating any lag time before observable current production. In addition, because the electrode is the only terminal electron acceptor for growth of *G. sulfurreducens* under these conditions, any increase in the planktonic optical density would be attributed to *R. palustris*.

**Figure 3:**
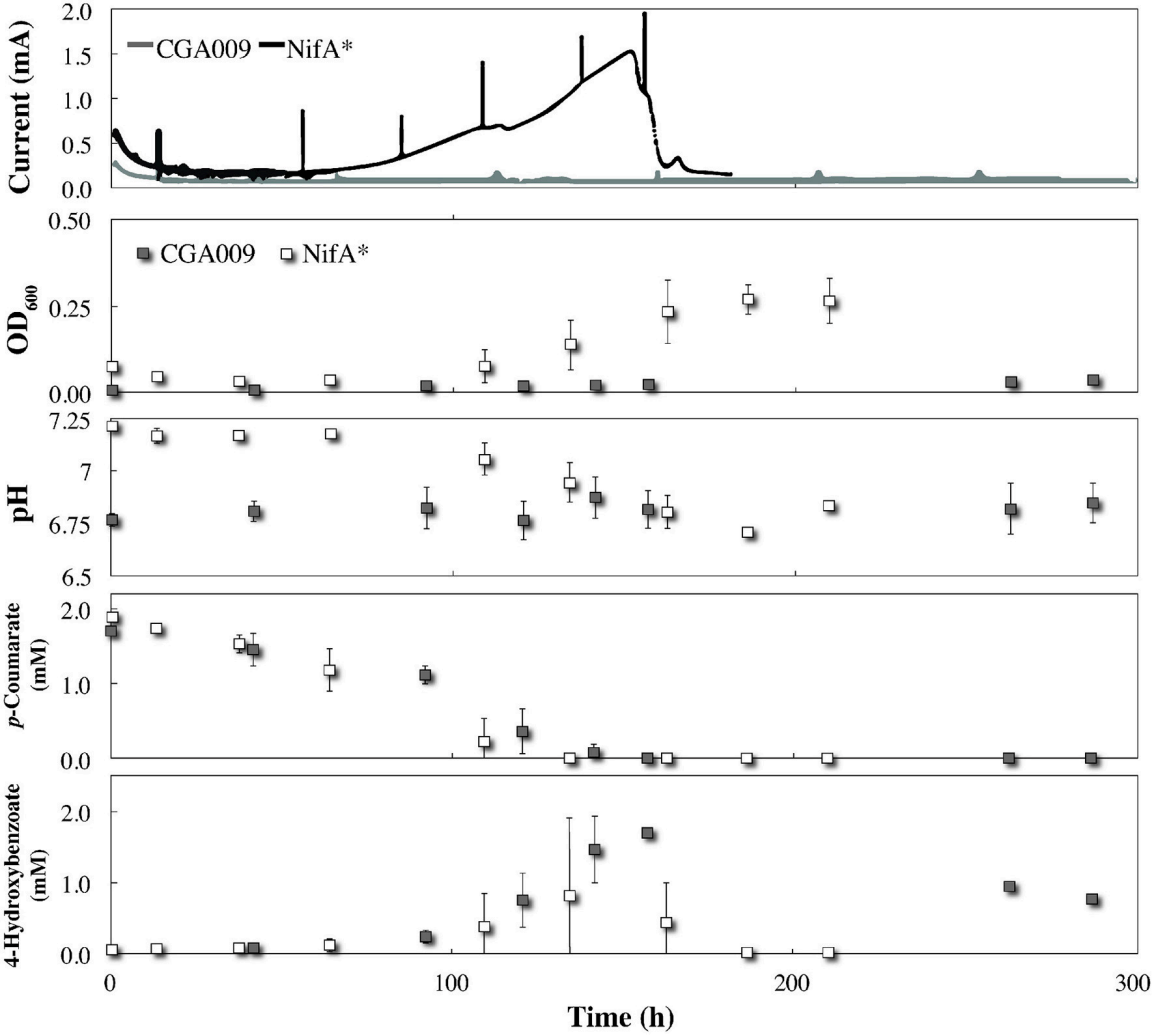
Co-culture growth of *R. palustris* CGA009 and NifA^*^ with *Geobacter sulfurreducens* in FW medium 2 mM *p*-coumarate with bicarbonate. Current for *R. palustris* CGA009 and NifA^*^ denoted by gray and black lines, respectively. Growth and metabolites for *R. palustris* CGA009 and NifA^*^ denoted by gray and white squares, respectively.

By comparing metabolite profiles with bicarbonate from the BESs (Figure 3) to those previously determined from serum bottle experiments (Figure 1), we found that conversion of *p*-coumarate to *p*-hydroxybenzoate and subsequent consumption occurred at nearly the same rate for both strains between both systems. However, the optical density for both strains was considerably lower in the BESs than when grown in serum bottles (Figures 1, 3). This was likely due to the preferential growth of *R. palustris* on the electrode and reactor surfaces compared to in the planktonic state since biofilm growth on all surfaces was observed in the BESs. Even with biofilm formation, the optical density trend with CGA009 growing at lower levels than NifA^*^, which we observed in serum bottles (Figure 1), was even more pronounced in the BESs (Figure 3).

While both strains appeared to perform similarly with regard to their metabolism of *p*-coumurate, NifA^*^ was the only strain of the two to elicit an electrochemical response from *G. sulfurreducens* in the BES. The electrochemical signal produced in the *R. palustris* NifA^*^ and *G. sulfurreducens* co-culture closely mirrored the metabolite profile, with current production beginning at the onset of *p*-coumarate consumption (Figure 3). Following the complete conversion of *p*-coumarate to 4-hydroxybenzoate, a short inflection in the current was observed while *R. palustris* transitioned to consuming 4-hydroxybenzoate (Figure 3). Finally, current production peaked at 1.43 ± 0.15 mA, which coincided with the decrease in measured 4-hydroxybenzoate concentrations (Figure 3). Collectively, this suggests that the observed current from the NifA^*^ *G. sulfurreducens* co-culture closely followed the metabolic trends of NifA^*^ growing in the electrochemical system, and was mediated by H_2_ evolution from the NifA^*^ strain rather than from acetate sharing since negligible current was produced from CGA009 under identical conditions (Figure 3). The lack of current produced from CGA009 likely demonstrates that no acetate was shared between *R. palustris* and *G. sulfurreducens* under these conditions, though, we cannot completely rule out the possibility that secreted acetate remained within the aggregations of *R. palustris* (Figure 2). On the other hand, from studies with dense anaerobic granules in bioreactors, we know that self-diffusion coefficients for granular biomass are 56–75% that of free water (Lens et al., 2003). With ample opportunities for diffusion of acetate out of the microbial aggregates and the ability of *G. sulfurreducens* to uptake acetate at μM levels (Esteve-Nunez et al., 2005), our observations strongly indicate that acetate sharing from the *p*-coumarate degrading population was not utilized as a strategy to aid redox balance under non-lethal conditions.

### Acetate sharing was not initiated even under lethal redox imbalance conditions

It is possible that acetate sharing in *R. palustris* may only be initiated under more stringent conditions, such as during a lethal redox-imbalance condition (without bicarbonate). In our BESs with *G. sulfurreducens*, we provide an efficient electron sink for acetate oxidation with an electrode. Thus, creating an ideal biological test bed in which stringent conditions can be combined with a possible solution to avoid a lethal redox imbalance—but only when acetate sharing is performed by *R. palustris*. To test this, *R. palustris* CGA009 and NifA^*^ were separately introduced into *G. sulfurreducens*-pregrown BESs with 2 mM *p*-coumarate FW medium without bicarbonate. To maintain a proper pH level and to neutralize H^+^, we included a 25 mM phosphate buffer. In addition, to provide an opportunity for association between *G. sulfurreducens* and *R. palustris* at the electrode, *R. palustris* CGA009 was pre-grown together with *G. sulfurreducens* on the electrode before switching to without bicarbonate conditions.

**Figure 4:**
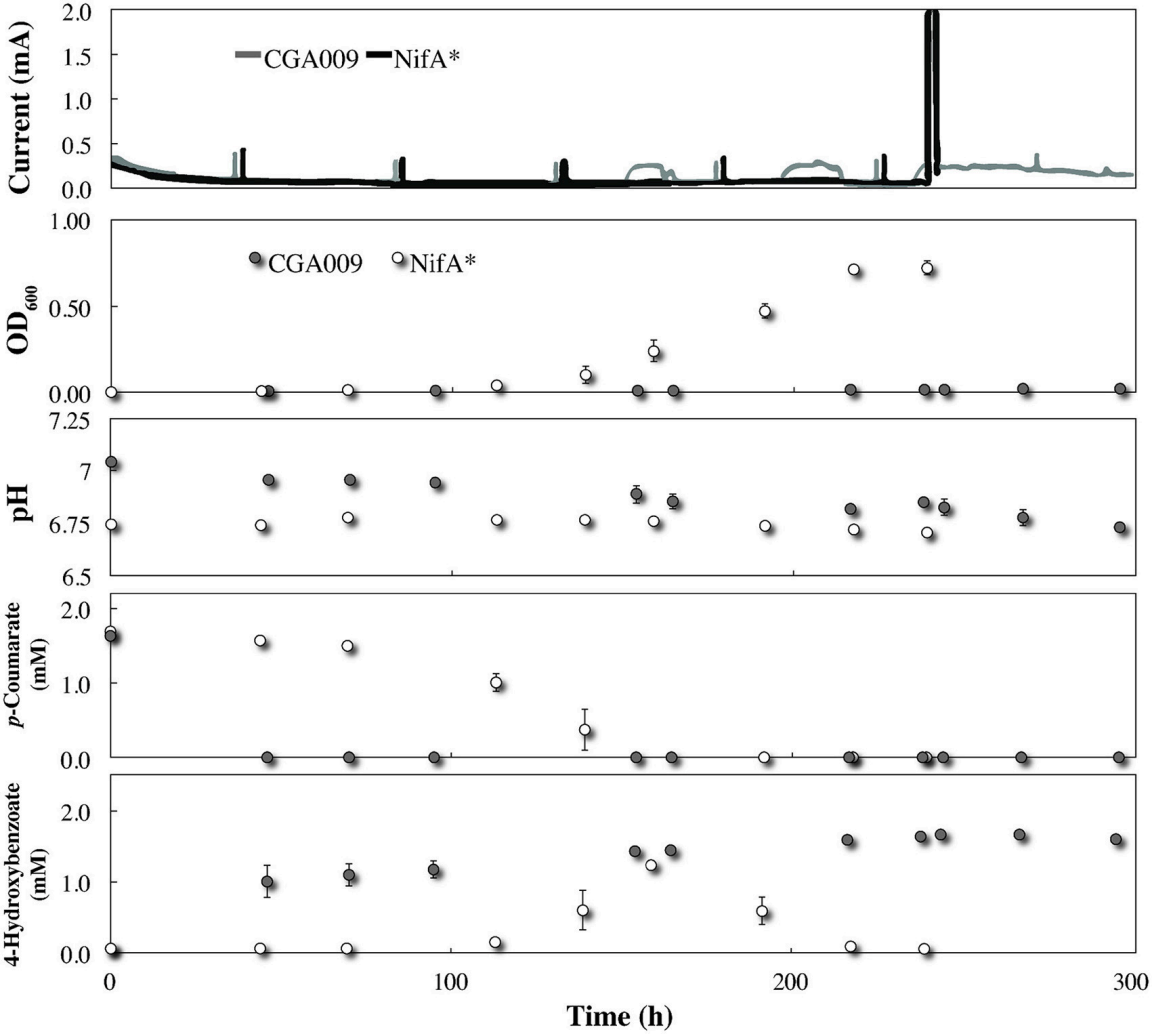
Co-culture growth of *R. palustris* CGA009 and NifA^*^ with *Geobacter sulfurreducens* in FW medium 2 mM *p*-coumarate without bicarbonate. *R. palustris* CGA009 and NifA^*^ are represented by the gray line or gray circles and black line or white circles, respectively. NifA^*^ was precultured in FW medium 2 mM *p*-coumarate without bicarbonte while CGA009 was pre-grown with *G. sulfurreducens* at the electrode.

Even though *R. palustris* CGA009 did not exhibit any planktonic growth within the BESs once conditions were switched to without bicarbonate, *p*-coumarate was rapidly taken up and metabolized to 4-hydroxybenozate after the medium replacement was completed (Figure 4). Because the acetyl-CoA unit derived from the conversion of *p*-coumarate to 4-hydroxybenzoate can be metabolized without producing net excess reducing equivalents (McKinlay and Harwood, 2010), it is expected that this conversion can be achieved without the aid from *G. sulfurreducens* at the electrode. Indeed, no electric current was registered during this time period (Figure 4). This conversion in the BES co-culture (Figure 4) occurred much faster than in the serum bottle cultures (Figure 1) due to the higher *R. palustris* biomass in the reactor from pre-culturing with *G. sulfurreducens*. Following the initial metabolism of *p*-coumarate to 4-hydroxybenzoate, no further metabolism was observed, resulting in a continued high concentration of this intermediate (Figure 4). This suggests that after consuming the alkyl moiety from *p*-coumarate, the excess of electrons from the aromatic group saturated the redox balance of *R. palustris* and growth was restricted. The absence of current production from *G. sulfurreducens* and inability of *R. palustris* to further catabolize the substrate demonstrates that even under restrictive growth conditions where redox imbalance becomes lethal, *R. palustris* CGA009 did not eliminate excess reducing equivalents in the form of acetate for oxidation by a syntrophic partner. Notable, however, when illumination was removed from the CGA009 culture without bicarbonate, a transient increase in electric current was observed without growth or further substrate metabolism (Figure 4 time: 150, 190, and 230 h). It is unclear to us why this relatively low electric current production (and possible acetate release) occurred in the co-culture during dark conditions and would require further investigation to discern.

Growth and metabolism kinetics of NifA^*^ in the co-culture without bicarbonate were very similar to our previous experiments including *p*-coumarate and 4-hydroxybenzoate (Figure 4). However, while NifA^*^ previously elicited a current response in FW medium and 2 mM *p*-coumarate with bicarbonate due to H_2_ production, no electric current was produced from growth of NifA^*^ with *G. sulfurreducens* without bicarbonate. This negative result identifies that acetate sharing was not induced with *G. sulfurreducens*. The lack of current produced by H_2_ oxidation by *G. sulfurreducens* was anticipated, since while it has been demonstrated that *G. sulfurreducens* can consume acetate and produce current in a phosphate buffer lacking bicarbonate, this has never been observed for just H_2_ (Soussan et al., 2013; Sun et al., 2014). To verify that this was a true negative result and rule out the possibility that *G. sulfurreducens* was simply inhibited following prolonged exposure to FW and 25 mM phosphate buffer without bicarbonate, sterile sodium acetate was injected into the BES at hour 240 after all *p*-coumarate had been consumed. We observed an immediate large electric current signal (current magnitude exceeded 2 mA scale) following the acetate addition, verifying that *G. sulfurreducens* was active (Figure 4).

**Figure 5:**
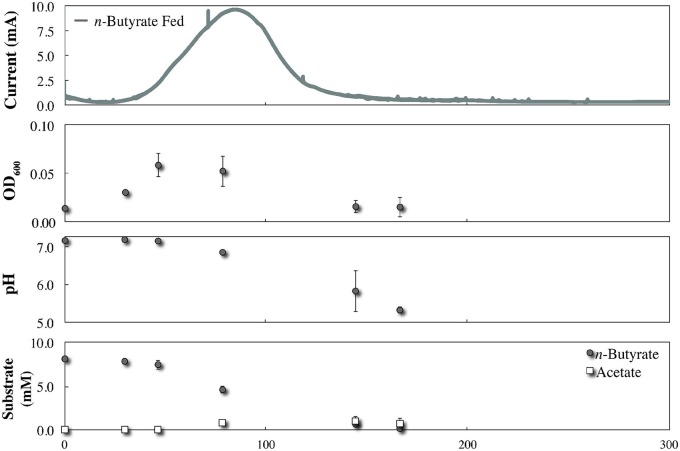
**Co-culture growth of *R. palustris* CGA009 with *Geobacter sulfurreducens* in FW medium with 11 mM *n*-butyrate and bicarbonate**.

During co-culture experiments we did not observe an acetate-based electric current signal from *G. sulfurreducens* as a product of the anaerobic degradation of *p*-coumarate by *R. palustris* (Figure 4). To validate that acetate released by *R. palustris* could in fact be utilized by *G. sulfurreducens* within this system, *R. palustris* CGA009 and *G. sulfurreducens* were co-cultured in FW with bicarbonate, and 2 mM *p*-coumarate was replaced with 11 mM *n*-butyrate. Because *G. sulfurreducens* cannot directly metabolize *n*-butyrate, and secretion of acetate had previously been measured during metabolism of *n*-butyrate by a pure culture of *R. palustris* (McKinlay and Harwood, 2011), this condition functions as a positive control for an acetate sharing-based electric signal (Figure 5). The production of a large current from within this system, and the emergence of a detectible concentration of acetate, coincides with butyrate consumption by *R. palustris* and validates our expectation that current would have been produced by *G. sulfurreducens* if acetate had been released by *R. palustris* during the metabolism of *p*-coumarate.

## Conclusion

The hypothesis that *R. palustris* engages in metabolite sharing within a single-genotype consortium to avoid complications of redox imbalance that can arise within a single cell was tested by investigating metabolite sharing of acetate between *R. palustris* and *G. sulfurreducens*. Since, *R. palustris* CGA009 cannot produce H_2_ when ${\text{NH}}_{4}^{+}$ is supplied as the only N source, *R. palustris* NifA^*^ was used as a redox balance positive control for growth and metabolite sharing (of H_2_) with *G. sulfurreducens*. An aggregating phenotype was initially observed for *R. palustris* CGA009, suggesting a desire to form close cellular interactions under these conditions, whereas NifA^*^ demonstrated no aggregating phenotype. This finding could have supported the acetate-sharing hypothesis, however, no current was produced by *G. sulfurreducens* through acetate sharing from *R. palustris* CGA009 within the BES under both non-lethal and lethal redox imbalance conditions. Although CGA009 was capable of complete metabolism of the alkyl side chain of *p*-coumarate, further metabolism of the aromatic group yielded too many reducing equivalents and quickly resulted in a lethal redox imbalance in BES conditions without bicarbonate. Because *G. sulfurreducens* functioned as the sole electron sink for oxidizing excess reducing equivalents by conversion of acetate to CO_2_ without bicarbonate, the absence of an acetate sharing-based electric current strongly indicates that *R. palustris* does not use acetate sharing to manage the excess of reducing equivalents *via* a single-genotype syntrophy. It was not expected that additional incubation of the CGA009 BES without bicarbonate would eventually stimulate acetate sharing with *G. sulfurreducens*, akin to adaptation phases in similar syntrophy evolution studies (Summers et al., 2010), since the acetate sharing activity would have already been present in the CGA009 preculture before the electrochemical experiment began.

## Author contributions

DD and LA designed the study; DD performed the research and sample analysis; DD analyzed the data, and DD and LA wrote the manuscript.

### Conflict of interest statement

The authors declare that the research was conducted in the absence of any commercial or financial relationships that could be construed as a potential conflict of interest.
